# Effectiveness and Tolerability of Repeated Courses of Viscosupplementation in Symptomatic Hip Osteoarthritis: A Retrospective Observational Cohort Study of High Molecular Weight *vs.* Medium Molecular Weight Hyaluronic Acid *vs.* No Viscosupplementation

**DOI:** 10.3389/fphar.2019.01007

**Published:** 2019-09-24

**Authors:** Orazio De Lucia, Luca Massimo Pierannunzii, Francesca Pregnolato, Elisa Verduci, Chiara Crotti, Elisabetta Valcamonica, Laura Pisoni, Daniela Comi, Paola Adele Lonati, Pier Luigi Meroni, Antonella Murgo

**Affiliations:** ^1^Department of Rheumatology and Medical Sciences, Clinical Rheumatology Unit, ASST Centro Traumatologico Ortopedico G. Pini-CTO, Milano, Italy; ^2^Department of Orthopedics, Sports Traumatology Unit, ASST Centro Traumatologico Ortopedico G. Pini-CTO, Milano, Italy; ^3^Immunorheumatology Research Laboratory, Istituto Auxologico Italiano, Milano, Italy; ^4^Department of Clinical and Experimental Medicine, Unit of Rheumatology, University of Messina, Messina, Italy; ^5^DISCCO-Department of Clinical Science and Community Health, Università degli Studi di Milano, Milano, Italy; ^6^Division of Internal Medicine and Cardiological Rehabilitation, Angelo Bellini Hospital - ASST Valle Olona, Somma Lombardo, Italy; ^7^Rheumatology Outpatient Clinic, ASST Nord Milano, Milano, Italy; ^8^Department of Rehabilitation, ASST Lecco, Lecco, Italy

**Keywords:** hyaluronic acid, hip osteoarthritis, viscosupplementation, VAS, WOMAC, hylan G-F 20, joint injection, ultrasound

## Abstract

**Background:** Nonsurgical management of symptomatic hip osteoarthritis needs real-world evidence. We evaluated the effectiveness and tolerability of US-guided intra-articular treatment of two hyaluronic acids (HAs) commercially available in Italy and investigated predictors of response.

**Methods:** Outpatient records including three cohorts: 122 subjects treated with medium (1,500–3,200 kDa; Hyalubrix^®^) molecular weight (MW) or high (hylan G-F20; Synvisc^®^) MW HAs and 20 controls taking NSAIDs/analgesics on demand were retrospectively analyzed. Pain VAS score, WOMAC, NSAID/analgesic consumption, and causes of suspension were available at 1, 6, 12, and 24 months after first administration. As selection bias usually affects observational retrospective studies, a quasi-randomization process was attained by performing propensity score approach.

**Results:** Propensity score adjustment successfully allowed comparisons among balanced groups of treatments. VAS and WOMAC considerably decreased over time in treated groups independently of the radiological grade (p<0.001). On the other hand, the control group showed only a slight and rather uneven variation in VAS. Mean score changes were comparable in both HA cohorts from the earliest stages (ΔVAS(HA1,500–3,200kDa)_T1vsT0_ = −20%; ΔVAS(hylan G-F20)_T1vsT0_ = −23%/ΔWOMAC(HA1,500–3,200kDa)_T1vsT0_ = −17%; ΔWOMAC(hylan G-F20)_T1vsT0_ = −19%), reaching a further substantial reduction after 12 months (ΔVAS(HA1,500–3,200kDa)_T12vsT0_ = −52%; ΔVAS(hylan G-F20)_T12vsT0_ = −53%/ΔWOMAC(HA1,500–3,200kDa)_T12vsT0_ = −45%; and ΔWOMAC(hylan G-F20)_T12vsT0_ = −47%). Almost 11% (=13/122) of ineffectiveness and few moderate local side effects 3% (=4/122) were detected.

**Conclusions:** Viscosupplementation in a real-life setting seems to provide a sound alternative in pain management in comparison to oral NSAIDs/analgesics, guaranteeing a reduced intake of pain killer medications. Analgesic effectiveness, functional recovery, and reduced joint stiffness extend and improve over 12 and 24 months, suggesting that repeated administrations achieve an additive effect.

## Introduction

Osteoarthritis (OA) is the most common cause of coxofemoral pain in adults, especially in elderly subjects (Hoaglund and Steinbach, 2001; Castell et al., 2015). First-line treatments are analgesics and non-steroidal anti-inflammatory drugs (NSAIDs) (Hochberg et al., 2012), along with rehabilitative physical therapy (Pisters et al., 2007; Hernandez-Molina et al., 2008). When patients do not benefit any more from conservative treatments, total joint arthroplasty is the last option. Surgical treatment is challenging, requires prolonged rehabilitation, and is burdened by serious risks of complications (infection, instability, deep vein thrombosis, etc.) (Tsertsvadze et al., 2014). Viscosupplementation is the injection of hyaluronic acid (HA) inside the ill joint in order to restore the physiological articular environment.

Despite the lack of evidence of HA efficacy in hip OA in 2000, in the following years, there were emerging evidence that it could be a treatment option (Migliore et al., 2003; Tikiz et al., 2005). The latest recommendations of Osteoarthritis Research Society International (OARSI) for the management of hip OA suggest intra-articular therapy with steroids and HA in addition to the standard therapy (Zhang et al., 2010). The hip is a difficult joint to inject, and many studies in the literature show greater safety and precision when the procedure is guided by ultrasound (US) (Migliore et al., 2003; Migliore et al., 2006b). Usually, many HAs with different characteristics are used (Migliore et al., 2016), but a clear priority is given to high molecular weight (MW) HA (Tikiz et al., 2005; van den Bekerom et al., 2008). Whether MW differences are associated with different therapeutic effects or durability, it is still to be clarified.

Aim of this study is to evaluate the effectiveness and tolerability of US-guided intra-articular treatment of symptomatic hip OA with two different HA commercially available in Italy (Synvisc^®^ 2 ml, a high MW HA, and Hyalubrix^®^ 2 ml, an intermediate MW HA) compared to standard analgesic/NSAID administration and to evaluate whether there are predictive parameters of response to treatment.

## Materials and Methods

This is a retrospective observational cohort study based on the patients’ database of a degenerative joint disease outpatient clinic.

Consecutive patients with hip OA who had received intra-articular HA injection were retrospectively reviewed.

### Selection Criteria

Patients were eligible for inclusion if they met the following criteria: symptomatic hip OA according to the American College of Rheumatology criteria (Altman et al., 1991); radiological grades II, III, and IV according to the Kellgren-Lawrence classification (Kellgren and Lawrence, 1957) evaluated by standard hip X-rays not older than 6 months before baseline (all the X-rays were interpreted by the same expert reader); and hip pain for at least 1 year. The included patients underwent viscosupplementation (treatment cohorts) or NSAID/analgesic administration on demand (control cohort) in case they rejected viscosupplementation (not willing to do it, fear of needles) with a minimum of 6-month follow-up between January 2006 and April 2014. Exclusion criteria were: patently secondary OA (after acetabular or cephalic fracture, avascular necrosis, developmental dysplasia, slipped capital femoral epiphysis, Legg-Calvè-Perthes disease, primary inflammatory rheumatic diseases), corticosteroid injection of the target hip in the previous 3 months, HA injection of the target hip in the previous year, previous open or arthroscopic surgery of the target hip, and ongoing systemic corticosteroid therapy. The patients were classified into three cohorts: HA 1,500–3,200 kDa, hylan G-F 20, and controls, depending on which HA was administered to them, if any.

### Hyaluronic Acid Features

HA 1,500–3,200 kDa 2ml (Hyalubrix^®^, Fidia Farmaceutici, Abano Terme [PD], Italy) is a sterile nonpyrogenic solution of HA sodium salt (15 mg/ml sodium HA) with a MW ranging between 1,500 and 3,200 kDa (medium MW). Hylan G-F 20 2ml (Synvisc^®^ Sanofi, Paris, France) is a sterile nonpyrogenic solution composed by chemically cross-linked hyaluronans (polyanionic form of hyaluronate), ranging between 4,000 and 6,000 kDa (high MW), termed hylans (hylan A soluble + hylan B insoluble gel).

### Viscosupplementation Dosing and Injection Procedure

**Figure 1:**
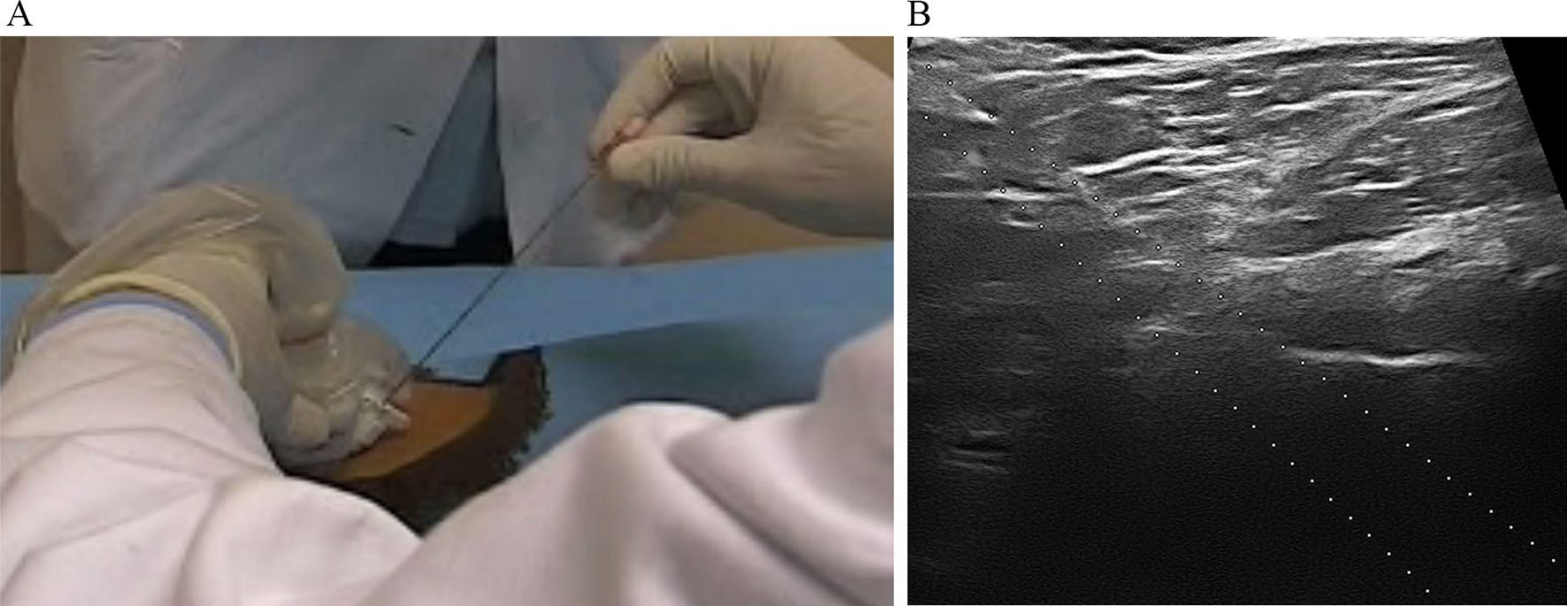
**(A)** Intra-articular injection performed by inserting a 18-gauge needle in the sterile guide with an antero-inferior approach; **(B)** intracapsular positioning of the needle was monitored in real time by direct US visualization of the needle on the screen of the ultrasound machine.

Each patient of viscosupplementation cohorts received three hip injections with HA 1,500–3,200 kDa 2 ml or hylan G-F 20 2 ml, once a month for three consecutive months, then further maintenance injections with the same HA were administered every 6 months for 2 years. All injections were carried out using a standardized technique, under US guidance. Through a 6–18 MHz linear transducer (Esaote MyLab 70) with a sterile guide attached, the hip joint was visualized using an anterior parasagittal scan, lateral to the femoral vessels. Intra-articular injection was performed by inserting a 18-gauge needle (15 cm long) in the sterile guide with an antero-inferior approach (**Figure 1A**) aiming at the top of the femoral head. Correct intracapsular positioning of the needle was monitored in real time by direct US visualization (**Figure 1B**).

### Data Collection

At baseline demographic data (age, gender, height, weight, and body mass index) duration of disease (calculated as time passed between the onset of symptoms and the pre-treatment data collection, termed “baseline” or t_0_), Kellgren-Lawrence radiological grade of OA, degree of hip pain reported on visual analogue scale (0–100 mm), WOMAC score (based on the three domains pain, stiffness, and joint function), and number of days of NSAID/analgesic consumption in the last month before visit for hip pain were collected.

At given time points after first injection (1, 6, 12, and 24 months), we gathered information about VAS score, WOMAC, number of days of NSAID/analgesic consumption, adverse events, and causes of treatment discontinuation.

### Data Analysis

Statistical analyses were performed with R software, version 3.0.2 for Windows [RStudio Team (2015). RStudio: Integrated Development for R. RStudio, Inc., Boston, MA URL http://www.rstudio.com/]. Descriptive statistics were used to summarize data. Overall, age, disease duration, and BMI were also coded as categorical factors (age: 0, if < 65 years; 1, otherwise; duration of disease: 0, if <5 years; 1, otherwise; BMI: 0, if <25; 1, otherwise). Associations and differences among the three treatments groups were assessed by chi-squared or Fisher’s exact tests and one-way ANOVA or Kruskal–Wallis tests, respectively. Cochrane–Armitage trend test was applied to compare percentage of patients using NSAIDs over time and among groups of treatment. Day-per-month consumption of NSAIDs/analgesics over time within the same group of treatment was assessed using Friedman test for repeated measurements and Dunn’s test for multiple comparisons adjustment. A p-value less or equal to 0.05 was considered statistically significant.

#### Quasi-Randomization Process

As observational studies can be affected by selection bias, propensity score (PS) approach (Rosenbaum and Rubin, 1983) was used for correcting the analysis of the nonrandomized design (D’Agostino, 1998). After generating a score based on the propensity for each patient of receiving a specific treatment given a set of baseline characteristics, the PS was included as covariate in the model further used to analyze repeated measures (Rosenbaum and Rubin, 1984). PS was calculated for each patient using logistic regression with treatment (HA or NSAIDs/analgesics) as dependent variable and baseline characteristics as independent variables. Covariates used for calculating PS were selected based on the method of standardized differences (Flury and Riedwyl, 1986) or considering characteristics at baseline that clearly differed among groups. A threshold of 0.10 of standard difference was considered as sign of important covariate imbalance.

#### Modeling Repeated Measures Over Time

The change over time of VAS and WOMAC scores, was assessed by the approach of generalized estimating equations (GEE) (Liang and Zeger, 1986) for repeated measurements with an exchangeable working correlation matrix. The variables of radiological grade of OA, administration timing, and therapy were included in the model in order to clarify their degree of association with outcomes and adjusted through PS.

Considering the small size of control group, bias-corrected estimates for the regression coefficients were calculated using a bias formula as provided by Lunardon et al. (Lunardon and Scharfstein, 2017).

### Compliance With Ethical Standards

The study was conducted according to the Declaration of Helsinki and approved by the local responsible Ethics Committee (Comitato Etico Milano Area B, protocol n° 125_2017). In this retrospective study, no written informed consent was required. Patient records/information were anonymized and de-identified prior to data analysis.

## Results

### Characteristics of the Study Population at Baseline

One hundred twenty-two patients (50 males and 72 females) with symptomatic hip OA met all the inclusion criteria: 117 with unilateral hip OA and 5 with bilateral hip OA who underwent bilateral viscosupplementation. Then, a total of 127 hips were treated. A cohort of 20 subjects (22 hips) managed with only NSAIDs/analgesics on demand served as controls.

The baseline characteristics of study population stratified by treatment are summarized in **Table 1**. In general, the three groups of patients turned out to be homogeneous in all demographic and clinical characteristics. Regarding disease scores, both HA groups showed a statistically significant difference compared to control group having a slightly higher WOMAC score, as shown in **Table 2** (left-hand column). On the other hand, the three groups did not differ in pain VAS score.

**Table 1:** Demographic and clinical characteristics of patients stratified by treatment, at time of recruitment. Continuous variables are summarized as mean ± SD, categorical variables as percentage (absolute frequency).

Baseline	Hyaluronic acid 1,500–3,200 kDa	HYLAN G-F 20	CTRLS	Overall p-value
	(N = 43)	(N = 79)	(N = 20)	
*Age (years)*	61.2 ± 14.8	63.0 ± 13.1	60.4 ± 11.1	0.647
*Age% (>65ys)*	39.5 (17)	50.6 (40)	40.0 (8)	0.429
*Gender F %(N)*	51.2 (22)	62.0 (49)	55.0 (11)	0.492
*Weight (kg)*	76.8 ± 12.7	72.6 ± 13.1	73.6 ± 10.2	0.215
*Height (cm)*	169.6 ± 9.9	166.2 ± 8.8	169.2 ± 7.4	0.105
*BMI (Kg/m^2^)*	26.6 ± 3.3	26.2 ± 3.6	25.7 ± 2.7	0.546
*BMI% (>25 Kg/m^2^*)	60.5 (26)	55.7 (44)	70.0 (14)	0.498
*Disease duration% (≥5ys)*	20.9 (9)	27.8 (22)	25.0 (5)	0.703
*K-L rating score %(N)*				0.322
Grade 2	32.6 (14)	22.8 (18)	15.0 (3)	
Grade 3	58.1 (25)	57.0 (45)	60.0 (12)	
Grade 4	9.3 (4)	20.3 (16)	25.0 (5)	

**Table 2:** Differences in VAS score and WOMAC index (mean ± SD) between patients treated with HA or controls at baseline before and after propensity score (PS) adjustment.

Comparison	Not adjusted difference	Difference after PS adjustment
**Hyaluronic acid 1,500–3,200 kDa *vs.* controls**		
*Pain VAS*	−1.3 ± 4.8 *(p = 0.783)*	−6.4 ± 4.8 *(p = 0.187)*
*WOMAC index*		
Total	8.2 ± 4.5 *(p = 0.07)*	2.7 ± 4.3 *(p = 0.537)*
Pain	2.0 ± 1.1 *(p = 0.07)*	0.9 ± 1.1 *(p = 0.435)*
Stiffness	1.4 ± 0.5 *(p = 0.008)*	0.7 ± 0.4 *(p = 0.071)*
Function	4.3 ± 3.5 *(p = 0.216)*	1.1 ± 3.4 *(p = 0.746)*
**HYLAN G-F 20 *vs.* NSAIDs**		
*Pain VAS*	1.8 ± 3.7 *(p = 0.638)*	−5.1 ± 4.0 *(p = 0.201)*
*WOMAC index*		
Total	10.3 ± 3.4 *(p = 0.002)*	2.6 ± 3.5 *(p = 0.466)*
Pain	2.5 ± 0.9 *(p = 0.006)*	0.9 + 1.0 *(p = 0.376)*
Stiffness	1.2 ± 0.5 *(p = 0.009)*	0.3 ± 0.3* (p = 0.320)*
Function	6.6 ± 2.6 *(p = 0.013)*	1.3 ± 2.8 *(p = 0.634)*

### Tolerability of Viscosupplementation

Overall, treatment with HAs was suspended in 20 out of 122 patients (16.4%), and the rate of suspension was similar (χ^2^ = 0.7, p = 0.400) between the two formulations (18.6% (8/43) *vs*. 15.2% (12/79), in HA 1,500–3,200 kDa and hylan G-F 20 groups, respectively). Reasons for HA 1,500–3,200 kDa suspension were related to side effects (n = 4 temporary worsening of hip pain recovering from 2 to 10 days) or ineffectiveness (n = 4), whereas reasons for hylan G-F 20 suspension were indicated as ineffectiveness (n = 9) or not reported (n = 3). Three out of four patients suspending because of side effects stopped the medication after 6 months while the last one complained hip pain after 1 year. Inefficacy occurred after 6 months or 1 year in 8 or 5 patients, respectively.

### Variation in NSAID/Analgesic Consumption During Follow-Up

At baseline, almost 80% of patients took NSAIDs/analgesics with an average consumption of about 10 days/month; the frequency of using it at baseline was comparable in the three groups of patients. During the 24 months of treatment with HA, a significant reduction in the use of NSAIDs/analgesics for pain control was observed both in terms of number of patients (HA 1,500–3,200 kDa *vs*. Ctrls, z = 3.64, p < 0.001; hylan G-F 20 *vs*. Ctrls, z = 4.25, p < 0.0001) (**Table 3**) and dosage (**Table 4**). In particular, HA-treated patients showed a significant decrease in day-per-month usage from the first month of therapy whereas, except for an increased consumption during the first month (p<0.01), controls maintained the same dosage over time.

**Table 3:** Percentages and relative frequencies (in brackets) of patients taking NSAID/analgesic medication in each group of treatment and observation period.

Therapy	Baseline	1 mth	6 mths	12mths	24 mths
Hyaluronic acid 1,500–3,200 kDa	83.7% (36/43)	53.5% (23/43)	58.1% (25/43)	38.9% (14/36)	17.1% (6/35)
					
Hylan G-F 20	77.2% (61/79)	57.0% (45/79)	50.6% (40/79)	44.2% (19/71)	48.0% (12/70)
					
Controls	75.0% (15/20)	70.0% (14/20)	85.0% (17/20)	80.0% (16/20)	90.0% (18/20)

**Table 4:** Mean values and related standard errors (SE) of NSAID/analgesic consumption (days/month) within each group of treatment.

Therapy	Mean (SE)	Overall p-value	p-value (t_i_ *vs.* t_0_)
**Hyaluronic acid 1,500–3,200 kDa**		<0.0001	
* Baseline*	9.7 (1.4)		−
* 1 mth*	5.0 (1.1)		0.003
* 6 mths*	4.5 (1.1)		0.0001
* 12 mths*	3.2 (0.9)		<0.0001
* 24 mths*	3.6 (1.0)		<0.0001
**Hylan G-F 20**		<0.0001	
* Baseline*	9.1 (1.1)		−
* 1 mth*	5.7 (1.0)		0.001
* 6 mths*	3.7 (0.7)		<0.0001
* 12 mths*	3.5 (0.7)		<0.0001
* 24 mths*	3.9 (0.7)		<0.0001
**Controls**		0.06	
* Baseline*	8.0 (1.8)		−
* 1 mth*	15.9 (2.4)		<0.01
* 6 mths*	11.5 (1.6)		0.157
* 12 mths*	11.4 (1.6)		0.152
* 24 mths*	11.7 (1.3)		0.091

### Propensity Score

Based on the standardized difference approach, the treatment groups were unbalanced in terms of age (st.diff. = 0.19), BMI (st. diff. = 0.24), baseline WOMAC pain (st.diff = 0.46), stiffness (st.diff. = 0.58), and function (st.diff. = 0.36), whilst balanced in terms of disease duration (st.diff. = 0.04) and VAS (st.diff. = 0.006). Then, PS was calculated using age, BMI, and all the baseline WOMAC domains as observed covariates. **Table 2** (right-hand column) shows improvement in the balance with regard to baseline WOMAC domains after PS adjustment.

### Variation of Pain VAS During Follow-Up

**Figure 2:**
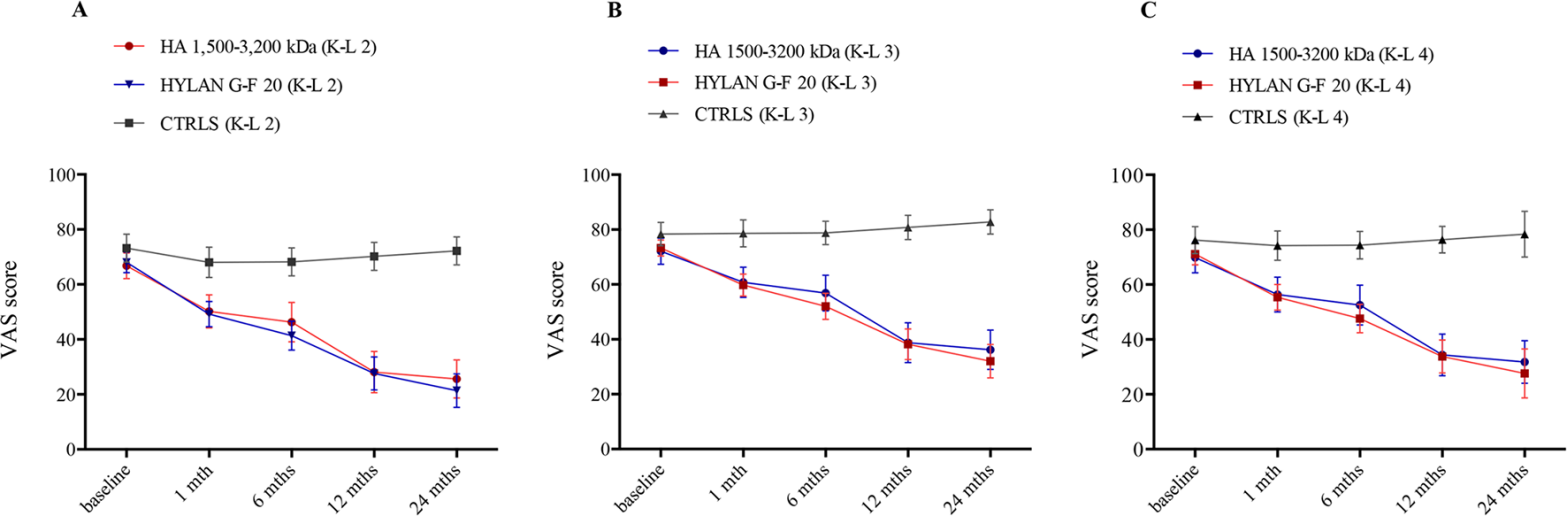
Time trend of VAS score over the course of follow-up (baseline and after 1, 6, 12, and 24 months) stratified by treatment (hyaluronic acid [HA] 1,500–3,200 kDa, hylan G-F 20, NSAIDS as controls [CTRLS]) and radiological grades II, III, and IV according to the Kellgren-Lawrence classification (**A**, K-L 2; **B**, K-L 3; **C**, K-L 4). Bars refer to standard error.

Patients treated with HAs clearly showed a different pathway in pain score when compared to controls during follow-up (**Figure 2**). **Table 5** shows the PS adjusted estimates [95% CI] of VAS scores at each time visit and stratified by radiological grade as resulted from GEE approach. Accordingly, patients undergoing HA treatments significantly decreased VAS score compared to baseline conditions since the first month of therapy and independently of radiological grade (i.e., the 95% CI of VAS variation for each K-L grade never include the null value). The progression in the decrease still occurred over time reaching a statistical significance after 12 months for both HAs (ΔVAS(HA 1,500–3,200 kDa)_T12vsT1_ = −24.2 [95% CI: −14.4 to −34.0], p<< 0.001; ΔVAS(hylan G-F 20)_ T12vsT1_: −23.8 [95% CI: −15.1 to −32.4], p<< 0.001) and was stable after 24 months as shown in **Figure 2**. When compared to controls, the effect of HA treatment was always significantly more relevant than NSAIDs/analgesics in all K-L grade subgroups (ΔVAS at 1-month follow-up: HA 1,500–3,200 kDa *vs.* Ctrls: −11.5 [95% CI: −18.9 to −4.1], p = 0.002; hylan G-F 20 *vs.* Ctrls: −13.7 [95% CI: −19.9 to −7.6], p<< 0.001). Overall, the trend of the two HAs overlapped almost perfectly.

**Table 5:** Follow-up variations of VAS score (mean [95% CI]) compared to baseline setting and stratified by radiological grade and therapy. All the estimates resulted by a quasi-randomization process obtained including propensity scores in the model for repeated measures (GEE model).

Timing x K-L grade	Hyaluronic acid 1,500–3,200 kDa	HYLAN G-F 20	Ctrls
**Grade II**			
* Baseline*	66.8 [57.5 to 75.9]	68.0 [60.6 to 75.4]	73.1 [63.3 to 83.1]
* 1 mth vs. baseline*	−16.6 [−22.3 to −10.9] p< < 0.001	−18.8 [−22.6 to −15.0] p< < 0.001	−5.1 [−9.8 to −0.4] p = 0.03
* 6 mths vs. baseline*	−20.5 [−29.7 to −11.3] p< < 0.001	−26.6 [−32.0 to −21.2] p< < 0.001	−4.9 [−8.5 to −1.2] p = 0.01
* 12 mths vs. baseline*	−38.7 [−48.7 to −28.7] p< < 0.001	−40.4 [−47.7 to −33.1] p< < 0.001	−2.9 [−5.7 to −0.1] p = 0.04
* 24 mths vs. baseline*	−41.2 [−51.3 to −31.1] p< < 0.001	−46.6 [−54.0 to -39.2] p< < 0.001	−0.9 [−4.2 to 2.3] p = 0.584
**Grade III**			
* Baseline*	72.1 [62.8 to 81.4]	73.3 [67.6 to 79.0]	78.4 [69.9 to 86.9]
* 1 mth vs. baseline*	−11.3 [−18.5 to −4.1] p = 0.004	−13.5 [−20.8 to −6.2] p = 0.001	0.2 [−7.6 to 8.0] p = 0.398
* 6 mths vs. baseline*	−15.2 [−24.6 to −5.8] p = 0.003	−21.3 [−29.7 to −12.9] p< < 0.001	0.4 [−6.7 to 7.5] P = 0.397
* 12 mths vs. baseline*	−33.3 [−43.7 to −22.9] p< < 0.001	−35.1 [−45.0 to −25.2] p< < 0.001	2.4 [−4.4 to 9.2] 0.315
* 24 mths vs. baseline*	−35.9 [−46.7 to −25.1] p< < 0.001	−41.3 [−50.8 to −31.8] p< < 0.001	4.4 [−2.5 to 11.3] p = 0.184
**Grade IV**			
* Baseline*	69.9 [59.0 to 80.8]	71.1 [63.6 to 78.6]	76.2 [66.6 to 85.8]
* 1 mth vs. baseline*	−13.5 [−22.6 to −4.4] p = 0.006	−15.7 [−24.5 to −6.9] p = 0.001	−2.0 [−11.1 to 7.1] p = 0.364
* 6 mths vs. baseline*	−17.4 [−28.4 to −6.4] p = 0.003	−23.5 [−32.8 to −14.2] p< < 0.001	−1.8 [−10.8 to 7.2] p = 0.369
* 12 mths vs. baseline*	−35.5 [−47.1 to −23.9] p< < 0.001	−37.3 [−48.1 to −26.5] p< < 0.001	0.2 [−8.2 to 8.6] p = 0.399
* 24 mths vs. baseline*	−38.1 [−50.0 to −26.2] p< < 0.001	−43.5 [−54.4 to −32.6] p< < 0.001	2.2 [−6.7 to 11.1] p = 0.355

As expected, we observed a significant slight effect of NSAIDs/analgesics on VAS score but only in patients reporting a K-L grade 2 (−5.1 [95% CI: −9.8 to −0.4], p = 0.03); such a change was gradually reduced over time as late as 12 months from the first visit (−2.9 [95% CI: −5.7 to −0.1], p = 0.04), then disappeared after 24 months (−0.9 [−4.2 to 2.3], p = 0.584).

### Variation of WOMAC During Follow-Up

**Figure 3:**
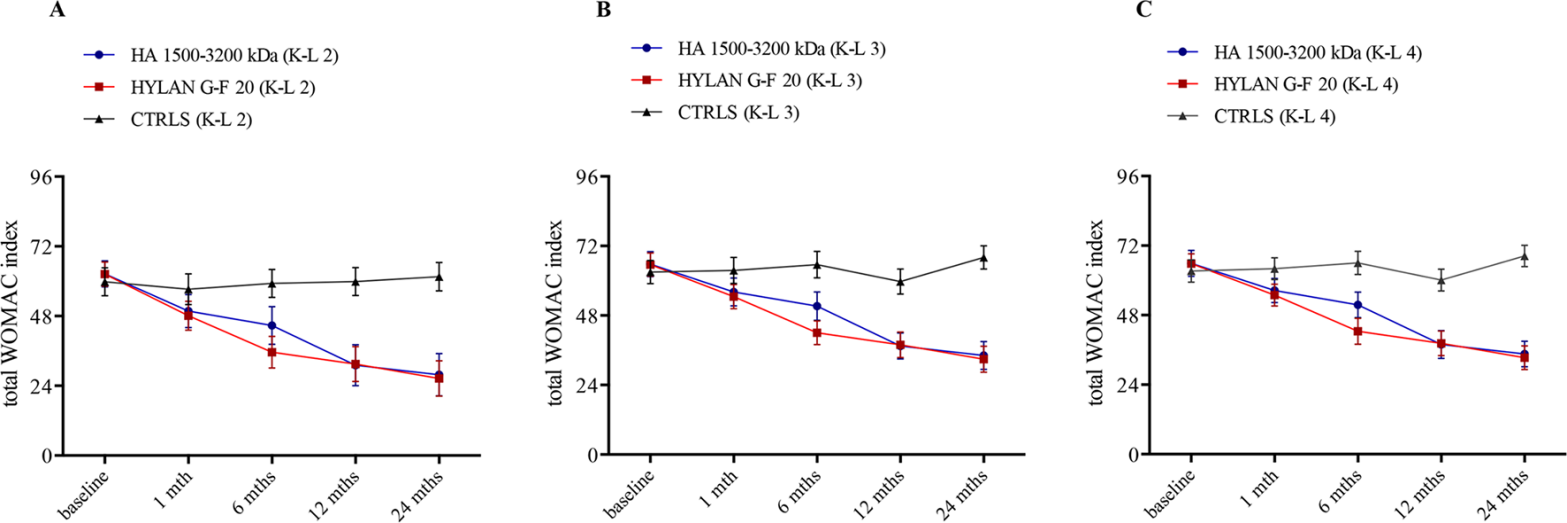
Time trend of total WOMAC index over the course of follow-up (baseline and after 1, 6, 12, and 24 months) stratified by treatment (hyaluronic acid [HA] 1,500–3,200 kDa, hylan G-F 20, NSAIDS as controls [CTRLS]) and radiological grades II, III, and IV according to the Kellgren-Lawrence classification (**A**, K-L 2; **B**, K-L 3; **C**, K-L 4). Bars refer to standard error.

The trend over time of WOMAC was investigated in terms of total score and its components, pain, stiffness and joint function domains. **Figure 3** illustrates the follow-up variations in total WOMAC stratified by treatment and K-L grade, and the related estimates are reported in **Table 6**. Both HAs significantly improved the total score compared to baseline conditions since the first month of therapy and independently of radiological grade (i.e., the 95% CI of VAS variation for each K-L grade never include the null value). An additional significant variation was found after 1 year of therapy (ΔWOMAC(HA 1,500–3,200 kDa)_T12vsT1_: −21.3 [95% CI: −12.6 to −30.0], p<< 0.001; ΔWOMAC(hylan G-F 20)_ T12vsT1_: −19.2 [95% CI: −10.8 to −27.6], p < 0.001) before finally setting in at the end of follow-up (**Figure 3**). Overall, the two HAs produced a similar trend in WOMAC change also after 6 months of therapy where hylan G-F 20 seemed to be more effective than HA 1,500–3,200 kDa (ΔWOMAC: −9.1 [95% CI: −18.7 to +0.4], p = 0.06).

**Table 6:** Follow-up variations in WOMAC score (mean [95% CI]) compared to baseline setting and stratified by radiological grade and therapy. All the estimates resulted by a quasi-randomization process obtained including propensity scores in the model for repeated measures (GEE model).

Timing x K-L grade	Hyaluronic acid 1,500–3,200 kDa	HYLAN G-F 20	Ctrls
**Grade II**			
* Baseline*	62.5 [53.7 to 71.3]	62.4 [54.2 to 70.6]	59.8 [50.4 to 69.2]
* 1 mth vs. baseline*	−12.8 [−17.9 to −7.7] p < 0.001	−14.3 [−19.4 to −9.2] p< < 0.001	−2.6 [−5.7 to 0.5] p = 0.100
* 6 mths vs. baseline*	−17.7 [−25.0 to −10.4] p< < 0.001	−26.8 [−32.9 to −20.7] p< < 0.001	−0.6 [−2.8 to 1.6] p = 0.593
* 12 mths vs. baseline*	−31.4 [−40.9 to −21.9] p< < 0.001	−30.9 [−38.2 to −23.6] p< < 0.001	+0.1 [1.3 to 0.0] p = 0.913
* 24 mths vs. baseline*	−34.7 [−44.7 to −24.7] p< < 0.001	−35.9 [−43.4 to −28.4] p< < 0.001	+1.8 [1.8 to 1.0] p = 0.312
**Grade III**			
* Baseline*	65.7 [57.3 to 74.1]	65.6 [57.7 to 73.5]	63.0 [55.2 to 70.8]
* 1 mth vs. baseline*	−9.6 [−16.4 to −2.8] p = 0.009	−11.1 [−19.1 to −3.1] p = 0.010	0.6 [−6.0 to 7.2] p = 0.393
* 6 mths vs. baseline*	−14.5 [−22.7 to −6.3] p = 0.001	−23.6 [−33.2 to −14.0] p< < 0.001	+2.6 [−3.3 to 8.5] p = 0.274
* 12 mths vs. baseline*	−28.2 [−38.6 to −17.8] p< < 0.001	−27.7 [−37.4 to −18.0] p< < 0.001	−3.3 −9.5 to 2.9] p = 0.233
* 24 mths vs. baseline*	−31.5 [−43.3 to −19.7] p< < 0.001	−32.7 [−41.1 to −24.3] p< < 0.001	+5.0 [−1.5 to 11.5] p = 0.129
**Grade IV**			
* Baseline*	65.9 [57.1 to 74.7]	65.8 [59.1 to 72.5]	63.2 [55.5 to 70.9]
* 1 mth vs. baseline*	−9.4 [−16.8 to −2.0] p = 0.019	−10.9 [−19.2 to −2.6] p = 0.014	+0.8 [−6.4 to 8.0] p = 0.389
* 6 mths vs. baseline*	−14.3 [−23.1 to −5.5] p = 0.002	−23.4 [−33.0 to −13.8] p< < 0.001	+2.8 [−4.0 to 9.6] p = 0.287
* 12 mths vs. baseline*	−28.0 [−38.3 to −17.7] p< < 0.001	−27.5 [−37.5 to −17.5] p< < 0.001	−3.1 [−10.1 to 3.9] p = 0.273
* 24 mths vs. baseline*	−31.3 [−42.4 to −20.2] p< < 0.001	−32.5 [−42.6 to −22.4] p< < 0.001	+5.2 [−2.0 to 12.4] p = 0.145

When compared to controls, the effect of HA treatment was always significantly more relevant than NSAIDs/analgesics in all K-L grade subgroups (ΔWOMAC at 1-month follow-up: HA 1,500–3,200 kDa *vs.* Ctrls: −10.2 [95% CI: −16.0 to −4.4], p < 0.001; hylan G-F 20 *vs.* Ctrls: −11.7 [95% CI: −17.6 to −5.9], p< < 0.001).

On the other hand, NSAIDs/analgesics did not show any effect at any time points and at any radiological grade (right-hand column of **Table 6**).

About WOMAC domains, the general trend of total score was confirmed by each component. In summary, patients undergoing HA treatment showed a remarkable effect since the first month of therapy in comparison with NSAID/analgesic group (*pain*: HA 1,500–3,200 kDa, T1 *vs.* T0: −2.3 [95% CI: −3.7 to −0.9], p = 0.001; hylan G-F 20, T1 *vs.* T0: −2.6 [95% CI: −4.0 to −1.2], p < 0.001; *stiffness*: HA 1,500–3,200 kDa, T1 *vs.* T0: −0.8 [95% CI: −1.4 to −0.1], p = 0.031; hylan G-F 20, T1 *vs.* T0: −0.7 [95% CI: −1.3 to −0.1]; *function*: HA 1,500–3,200 kDa, T1 *vs.* T0: −7.1 [95% CI: −11.3 to −2.9], p < 0.001; hylan G-F 20, T1 *vs.* T0: −8.5 [95% CI: −12.8 to −4.1], p < 0.001). The baseline scores of the three K-L grades were comparable within each domain (*pain*: K-L 3 *vs.* K-L 2: +1.2 [95% CI: −0.2 to 2.6], p = 0.09, and K-L 4 *vs.* K-L 2: +1.0 [95% CI: −0.8 to 2.8], p = 0.271; *function*: K-L 3 *vs.* K-L 2: +1.8 [95% CI: −2.8 to 6.4], p = 0.445 and K-L 4 *vs.* K-L 2: +2.0 [95% CI: −4.0 to 8.0], p = 0.515) except for stiffness where patients with radiological grade 4 reported a slightly greater WOMAC score compared to patients with radiological grade 2 (K-L 3 *vs.* K-L 2: +0.3 [95% CI: −0.2 to 0.8], p = 0.228, and K-L 4 *vs.* K-L 2: +0.8 [95% CI: 0.1 to 1.4] score units, p = 0.021).

Furthermore, the pattern of pain domain in controls was consistent with the VAS score variations since they showed a mild, even if not significant, improvement during the first two visits (month 1: −0.7 [95% CI: −1.6 to 0.1] score units, p = 0.085; month 2: −0.6 [−1.2 to 0.06], p = 0.075).

## Discussion

### Comments on the Effectiveness and Safety

The primary endpoint of this study was to evaluate the effectiveness of viscosupplementation expressed as a reduction in VAS and WOMAC scores. Our data confirmed the analgesic effectiveness of viscosupplementation in a statistically significant way after every administration for both HAs. The percentage of VAS reduction was around 20–30%, in accordance with the literature (Migliore et al., 2011b). After the first month of treatment, patients treated with HAs reported a lower VAS score than patients treated with NSAIDs/analgesics alone. This reduction was not only maintained over time but was further reduced upon each following administration leading to a decrease of 50–70% at the end of follow-up. To the best of our knowledge, this information is novel for hip OA; this finding further supports the reported ability of retreatment to consolidate single injections highlighted so far in knee OA (Navarro-Sarabia et al., 2011; Raman et al., 2018).

The overall effectiveness of the therapy over 2 years of treatment with high MW HA was similar to that obtained with intermediate MW HA: this confirms the observations of the studies published thus far comparing HAs with different MW (Tikiz et al., 2005; van den Bekerom et al., 2008).

HA safety profile confirms the positive data already present in the literature (Migliore et al., 2011a) and indicates that HA injections reduce NSAID/analgesic intake, possibly avoiding the most frequent complications linked to these drugs (such as gastrointestinal/renal complications and increased cardiovascular mortality) (Garcia Rodriguez and Hernandez-Diaz, 2001; Crofford, 2013).

### Confounding Factors

Direct involvement in pain perception has not been demonstrated for any demographic and clinical variables; however, these factors may have a confounding role, and therefore, it is essential to take them into consideration estimating the VAS and WOMAC variation under HA treatments. Indeed, a different distribution of these characteristics among groups of treatment can be source of unbalanced designs in retrospective studies. Thus, we successfully performed a PS analysis to address selection bias due to non-random assignment of patients to treatment (**Table 2**).

Furthermore, radiological grade was investigated as prognostic factor of response to treatment. Presumably, the highest radiological grade may lead to an even poorer response to treatment because it is more difficult to treat chronic pain due to adaptive circuits of the nervous system (Lee et al., 2011). However, in our cohort, radiological grade was not mainly involved in response to treatment, and the effectiveness of HAs was always relevant independently of severity of disease both in terms of VAS and WOMAC scores.

Our data, together with the encouraging results obtained in various studies on the reduction of progression toward total hip arthroplasty (Migliore et al., 2012a; Migliore et al., 2012b; Tsertsvadze et al., 2014), support the use of viscosupplementation also in patients with severe hip OA when total hip arthroplasty is not feasible or refused.

### Merits and Flaws of the Study

This study has several strengths. The sample size is rather large compared to other similar studies, and the observation period is longer than the majority of other studies in the literature, which usually have a follow-up ranging between 6 and 18 months (Migliore et al., 2006a; Migliore et al., 2006b; Migliore et al., 2011b; Rivera, 2016). Recently, data from ANTIAGE registry including more than 1,000 hip OA patients were analyzed and published (Migliore et al., 2017), but the study lacks completely a control group that is a significant criterion for conducting a study in evidence-based medical research.

Furthermore, we achieved balanced groups of treatment because of the PS approach; therefore, we reduced bias due to confounding factors and correctly estimated the effect of therapy by accounting for the covariates that predict the receiving treatment. PS analysis resulted particularly efficient since our population was naturally quite homogeneous for most demographic and clinical characteristics. Indeed, control group included patients that spontaneously rejected viscosupplementation, meaning that treatment allocation was not a clinician’s choice.

On the other hand, the limitations of this statistical analysis should not be forgotten. In fact, PS does not correct for unobservable or unmeasured variables.

Finally, it should be highlighted that the treatment effect was estimated performing a GEE model that handles missing responses due to treatment discontinuation (side effects or ineffectiveness).

A limitation of this study consists in the few numbers of patients treated with NSAIDs/analgesics. Undeniably, control group was numerically less represented than HA groups. In order to address this issue, we applied a correction to regression coefficients of GEE model. However, the choice and the enrolment of a control group in clinical practice of hip pain management is challenging as reported also in the mini-review of Migliore and Anichini (2017).

Finally, we observed an improved pain score as measured by standardized and feasible tools such as VAS and WOMAC indexes. Although these outcomes allow a direct feedback as reported by patients themselves, additional efforts should be made to accomplish a whole evaluation, as recently suggested by Migliore and colleagues (2015).

## Conclusions

US-guided hip injection technique allows us to act safely and accurately, without exposing the patient to ionizing radiation. High MW and medium MW HAs are both effective in the reduction of VAS and WOMAC at all the time intervals considered in patients with hip OA.

Our findings suggest that US-guided intra-articular HA injection is an effective and well tolerated treatment by patients with symptomatic hip OA. The benefits, such as pain reduction, functional recovery, and reduced joint stiffness, extend and improve over 12 months from the first injection, suggesting that repeated administrations display an additive effect. Moreover, we showed that HA injection is effective against pain beginning with the first administration.

Interestingly, a reduction in NSAID/analgesic intake is confirmed with viscosupplementation, with further potential benefits for general health of patients, tapering NSAID consumption and lowering classical drug side effects (Garcia Rodriguez and Hernandez-Diaz, 2001; Crofford, 2013).

Larger and longer prospective studies are needed to better estimate the effect of HA therapy in OA patients with different demographic and clinical characteristics. Unbiased outcomes as X-ray performed at the end of the treatment or the measure of the time to prosthesis should be considered (Migliore et al., 2015).

## Ethics Statement

The study protocol was planned according to the Declaration of Helsinki and was approved by the local responsible Ethics Committee (protocol n° 125_2017).

## Author Contributions

Substantial contributions to study conception and design: OL, AM. Substantial contributions to acquisition of data: OL, LMP, EVe, CC, EVa, LP, DC, AM. Substantial contributions to analysis and interpretation of data: FP, OL, EVe, PL, PM, AM. Drafting the article or revising it critically for important intellectual content: all the authors. Final approval of the version of the article to be published: all the authors.

## Conflict of Interest Statement

The authors declare that the research was conducted in the absence of any commercial or financial relationships that could be construed as a potential conflict of interest.

The reviewer OM declared a shared affiliation, with no collaboration, with one of the authors, CC, to the handling editor at the time of the review.

## References

[B1] Altman R. Alarcon G. Appelrouth D. Bloch D. Borenstein D. Brandt K. (1991). The American college of rheumatology criteria for the classification and reporting of osteoarthritis of the hip. Arthritis Rheum. 34 (5), 505–514. 10.1002/art.1780340502 2025304

[B2] Castell M. V. van der Pas S. Otero A. Siviero P. Dennison E. Denkinger M. (2015). Osteoarthritis and frailty in elderly individuals across six European countries: results from the European Project on OSteoArthritis (EPOSA). BMC Musculoskelet. Disord. 16, 359. 10.1186/s12891-015-0807-8 26578262 PMC4650343

[B3] Crofford L. J. (2013). Use of NSAIDs in treating patients with arthritis. Arthritis Res. Ther. 15 Suppl 3, S2. 10.1186/ar4174 PMC3891482 24267197

[B4] D’Agostino R. B. Jr. (1998). Propensity score methods for bias reduction in the comparison of a treatment to a non-randomized control group. Stat. Med. 17 (19), 2265–2281. 10.1002/(SICI)1097-0258(19981015)17:19<2265::AID-SIM918>3.0.CO;2-B 9802183

[B5] Flury B. K. Riedwyl H. (1986). Standard distance in univariate and multivariate analysis. Am. Stat. 40, 249–251. 10.1080/00031305.1986.10475403

[B6] Garcia Rodriguez L. A. Hernandez-Diaz S. (2001). The risk of upper gastrointestinal complications associated with nonsteroidal anti-inflammatory drugs, glucocorticoids, acetaminophen, and combinations of these agents. Arthritis Res. 3 (2), 98–101. 10.1186/ar146 11178116 PMC128885

[B7] Hernandez-Molina G. Reichenbach S. Zhang B. Lavalley M. Felson D. T. (2008). Effect of therapeutic exercise for hip osteoarthritis pain: results of a meta-analysis. Arthritis Rheum. 59 (9), 1221–1228. 10.1002/art.24010 18759315 PMC2758534

[B8] Hoaglund F. T. Steinbach L. S. (2001). Primary osteoarthritis of the hip: etiology and epidemiology. J. Am. Acad. Orthop. Surg. 9 (5), 320–327. 10.5435/00124635-200109000-00005 11575911

[B9] Hochberg M. C. Altman R. D. April K. T. Benkhalti M. Guyatt G. McGowan J. (2012). American college of rheumatology 2012 recommendations for the use of nonpharmacologic and pharmacologic therapies in osteoarthritis of the hand, hip, and knee. Arthritis Care Res. (Hoboken) 64 (4), 465–474. 10.1002/acr.21596 22563589

[B10] Kellgren J. H. Lawrence J. S. (1957). Radiological assessment of osteo-arthrosis. Ann. Rheum. Dis. 16 (4), 494–502. 10.1136/ard.16.4.494 13498604 PMC1006995

[B11] Lee Y. C. Nassikas N. J. Clauw D. J. (2011). The role of the central nervous system in the generation and maintenance of chronic pain in rheumatoid arthritis, osteoarthritis and fibromyalgia. Arthritis Res. Ther. 13 (2), 211. 10.1186/ar3306 21542893 PMC3132050

[B12] Liang K. Y. Zeger S. L. (1986). Longitudinal data analysis using generalized linear models. Biometrika 73 (1), 13–22. 10.1093/biomet/73.1.13

[B13] Lunardon N. Scharfstein D. (2017). Comment on ‘small sample GEE estimation of regression parameters for longitudinal data’. Stat. Med. 36 (22), 3596–3600. 10.1002/sim.7366 28868672

[B14] Migliore A. Bella A. Bisignani M. Calderaro M. De Amicis D. Logroscino G. (2012a). Total hip replacement rate in a cohort of patients affected by symptomatic hip osteoarthritis following intra-articular sodium hyaluronate (MW 1,500-2,000 kDa) ORTOBRIX study. Clin. Rheumatol. 31 (8), 1187–1196. 10.1007/s10067-012-1994-4 22678146

[B15] Migliore A. Bizzi E. De Lucia O. Sedie A. D. Tropea S. Bentivegna M. (2016). Differences regarding branded HA in Italy, part 2: data from clinical studies on knee, hip, shoulder, Ankle, temporomandibular joint, vertebral facets, and carpometacarpal joint. Clin. Med. Insights Arthritis Musculoskelet. Disord. 9, 117–131. 10.4137/CMAMD.S39143 27279754 PMC4898442

[B16] Migliore A. Bizzi E. Herrero-Beaumont J. Petrella R. J. Raman R. Chevalier X. (2015). The discrepancy between recommendations and clinical practice for viscosupplementation in osteoarthritis: mind the gap! Eur. Rev. Med. Pharmacol. Sci. 19 (7), 1124–1129. 25912569

[B17] Migliore A. Bizzi E. Massafra U. Bella A. Piscitelli P. Lagana B. (2012b). The impact of treatment with hylan G-F 20 on progression to total hip arthroplasty in patients with symptomatic hip OA: a retrospective study. Curr. Med. Res. Opin. 28 (5), 755–760. 10.1185/03007995.2011.645563 22126424

[B18] Migliore A. Granata M. Tormenta S. Lagana B. Piscitelli P. Bizzi E. (2011a). Hip viscosupplementation under ultra-sound guidance riduces NSAID consumption in symptomatic hip osteoarthritis patients in a long follow-up. Data from Italian registry. Eur. Rev. Med. Pharmacol. Sci. 15 (1), 25–34. 21381497

[B19] Migliore A. Martin L. S. Alimonti A. Valente C. Tormenta S. (2003). Efficacy and safety of viscosupplementation by ultrasound-guided intra-articular injection in osteoarthritis of the hip. Osteoarthr. Cartil. 11 (4), 305–306. 10.1016/S1063-4584(03)00008-6 12681959

[B20] Migliore A. Massafra U. Bizzi E. Lagana B. Germano V. Piscitelli P. (2011b). Intra-articular injection of hyaluronic acid (MW 1,500-2,000 kDa; HyalOne) in symptomatic osteoarthritis of the hip: a prospective cohort study. Arch. Orthop. Trauma Surg. 131 (12), 1677–1685. 10.1007/s00402-011-1353-y 21814776

[B21] Migliore A. Massafra U. Frediani B. Bizzi E. Sinelnikov Yzchaki E. Gigliucci G. (2017). HyalOne(R) in the treatment of symptomatic hip OA—data from the ANTIAGE register: seven years of observation. Eur. Rev. Med. Pharmacol. Sci. 21 (7), 1635–1644. 28429341

[B22] Migliore A. Tormenta S. Martin Martin L. S. Iannessi F. Massafra U. Carloni E. (2006a). The symptomatic effects of intra-articular administration of hylan G-F 20 on osteoarthritis of the hip: clinical data of 6 months follow-up. Clin. Rheumatol. 25 (3), 389–393. 10.1007/s10067-005-0052-x 16249827

[B23] Migliore A. Tormenta S. Massafra U. Martin Martin L. S. Carloni E. Padalino C. (2006b). [18-month observational study on efficacy of intraarticular hyaluronic acid (hylan G-F 20) injections under ultrasound guidance in hip osteoarthritis]. Reumatismo 58 (1), 39–49. 10.4081/reumatismo.2006.39 16639487

[B24] Navarro-Sarabia F. Coronel P. Collantes E. Navarro F. J. de la Serna A. R. Naranjo A. (2011). A 40-month multicentre, randomised placebo-controlled study to assess the efficacy and carry-over effect of repeated intra-articular injections of hyaluronic acid in knee osteoarthritis: the AMELIA project. Ann. Rheum. Dis. 70 (11), 1957–1962. 10.1136/ard.2011.152017 21852252 PMC3184238

[B25] Pisters M. F. Veenhof C. van Meeteren N. L. Ostelo R. W. de Bakker D. H. Schellevis F. G. (2007). Long-term effectiveness of exercise therapy in patients with osteoarthritis of the hip or knee: a systematic review. Arthritis Rheum. 57 (7), 1245–1253. 10.1002/art.23009 17907210

[B26] Raman R. Henrotin Y. Chevalier X. Migliore A. Jerosch J. Monfort J. (2018). Decision algorithms for the retreatment with viscosupplementation in patients suffering from knee osteoarthritis: recommendations from the EUROpean VIScosupplementation COnsensus Group (EUROVISCO). Cartilage 9 (3), 263–275. 10.1177/1947603517693043 29110511 PMC6042033

[B27] Rivera F. (2016). Single intra-articular injection of high molecular weight hyaluronic acid for hip osteoarthritis. J. Orthop. Traumatol. 17 (1), 21–26. 10.1007/s10195-015-0381-8 26449357 PMC4805628

[B28] Rosenbaum P. R. Rubin D. B. (1983). The central role of the propensity score in observational studies for causal effects. Biometrika 70, 41–55. 10.1093/biomet/70.1.41

[B29] Rosenbaum P. R. Rubin D. B. (1984). Reducing bias in observational studies using subclassification on the propensity score. J. Am. Stat. Assoc. 79, 516–524. 10.1080/01621459.1984.10478078

[B30] Tikiz C. Unlu Z. Sener A. Efe M. Tuzun C. (2005). Comparison of the efficacy of lower and higher molecular weight viscosupplementation in the treatment of hip osteoarthritis. Clin. Rheumatol. 24 (3), 244–250. 10.1007/s10067-004-1013-5 15647968

[B31] Tsertsvadze A. Grove A. Freeman K. Court R. Johnson S. Connock M. (2014). Total hip replacement for the treatment of end stage arthritis of the hip: a systematic review and meta-analysis. PLoS One 9 (7), e99804. 10.1371/journal.pone.0099804 25003202 PMC4086719

[B32] van den Bekerom M. P. Rys B. Mulier M. (2008). Viscosupplementation in the hip: evaluation of hyaluronic acid formulations. Arch. Orthop. Trauma Surg. 128 (3), 275–280. 10.1007/s00402-007-0374-z 17572901 PMC2228384

[B33] Zhang W. Nuki G. Moskowitz R. W. Abramson S. Altman R. D. Arden N. K. (2010). OARSI recommendations for the management of hip and knee osteoarthritis: part III: changes in evidence following systematic cumulative update of research published through January 2009. Osteoarthr. Cartil. 18 (4), 476–499. 10.1016/j.joca.2010.01.013 20170770

